# Humanized DRAGA mice are a valuable model to study novel immunotherapies for HIV-1

**DOI:** 10.1093/jimmun/vkaf185

**Published:** 2025-08-13

**Authors:** Pongthorn Pumtang-On, Liliana K Thron, Negin Goodarzi, Brianna C Davey, Emily N Sevcik, Natalie Coleman-Fuller, Ahmad F Karim, Vaiva Vezys, Mangala Rao, Branden S Moriarity, Mary S Pampusch, Aaron K Rendahl, Sofia A Casares, Pamela J Skinner

**Affiliations:** Department of Veterinary and Biomedical Sciences, University of Minnesota, St Paul, MN, United States; Department of Veterinary and Biomedical Sciences, University of Minnesota, St Paul, MN, United States; Graduate Program in Molecular Pharmacology and Therapeutics, University of Minnesota, Minneapolis, MN, United States; Department of Veterinary and Biomedical Sciences, University of Minnesota, St Paul, MN, United States; Department of Veterinary and Biomedical Sciences, University of Minnesota, St Paul, MN, United States; Department of Veterinary and Biomedical Sciences, University of Minnesota, St Paul, MN, United States; MarPam Pharma, LLC, St Paul, MN, United States; Agile Vaccines and Therapeutic, Infectious Diseases Directorate, Naval Medical Research Command, Silver Spring, MD, United States; Center for Immunology, Department of Microbiology and Immunology, University of Minnesota, Minneapolis, MN, United States; Laboratory of Adjuvant and Antigen Research, United States Military HIV Research Program, Walter Reed Army Institute of Research, Silver Spring, MD, United States; Department of Pediatrics, University of Minnesota, Minneapolis, MN, United States; Department of Veterinary and Biomedical Sciences, University of Minnesota, St Paul, MN, United States; Department of Veterinary and Biomedical Sciences, University of Minnesota, St Paul, MN, United States; Agile Vaccines and Therapeutic, Infectious Diseases Directorate, Naval Medical Research Command, Silver Spring, MD, United States; Department of Veterinary and Biomedical Sciences, University of Minnesota, St Paul, MN, United States; MarPam Pharma, LLC, St Paul, MN, United States

**Keywords:** ART, B-cell follicles, CAR/CXCR5 T cells, HIV, humanized DRAGA mice

## Abstract

Humanized (h) DRAGA mice are a promising in vivo model for investigating immunotherapies for treating HIV infections. These mice are not only susceptible to HIV infection, but they also develop functional human immune cells, including T cells and B cells, as well as follicular-like structures that mimic lymphoid B-cell follicles, where HIV-producing cells concentrate during infection in a manner similar to that found in humans. This study evaluated HIV-infected hDRAGA mice as a model for testing the safety, tissue targeting, and efficacy of HIV-specific CAR/CXCR5 T cells. We also evaluated whether HIV infection in hDRAGA mice can be suppressed by antiretroviral therapy. We produced functional HIV-specific CAR/CXCR5 T cells from disaggregated hDRAGA splenocytes and infused cell products into HIV-infected hDRAGA mice. CAR/CXCR5 T cells persisted in hDRAGA mice for the duration of the study, peaking 6 d postinfusion. Treatment with CAR/CXCR5 T cells appeared to be safe, with 100% survival rate and no noticeable changes in pathology. Six days after infusion, CAR/CXCR5 T cells had accumulated in the follicle-like structures in the spleen, with many in direct contact with HIV-producing cells. However, CAR/CXCR5 T-cell treatment did not reduce viral loads compared to controls, likely because CD4 T cells in the infused product became infected with and spread HIV infection. Despite this, all mice treated with antiretroviral therapy showed complete suppression of viral replication, indicating that HIV infection was treatment responsive in the DRAGA mice. These studies indicate that hDRAGA mice are a valuable model to study cellular immunotherapies for HIV.

## Introduction

In 2022, it was estimated that 39 million people were living with HIV worldwide, with 1.3 million people acquiring HIV infection during the year.^1^ Although antiretroviral therapy (ART) is effective in suppressing HIV, it is not a cure. Upon cessation of ART, viremia ensues. Thus, there is a need to create improved therapies for HIV infections that lead to sustained long-term virologic control.

Chimeric antigen receptor (CAR) T cells are a promising therapy for targeting HIV, as they can be engineered to be directly cytotoxic to cells expressing specific viral targets on their surfaces.^2^ First introduced in the field of cancer research, CAR T cells have been used to successfully treat B-cell malignancies.^3–6^ We developed HIV- and simian immunodeficiency virus (SIV)–specific CAR/CXCR5 T cells containing a bispecific CD4-mannose-binding lectin (MBL) CAR^7^ and encoding a follicular homing receptor (CXCR5) to target B-cell follicles.^8^^,^^9^ HIV- and SIV-specific CAR/CXCR5 T cells showed specific cytotoxic functions and suppression in vitro.^7–9^ In an SIV-infected rhesus macaque model of HIV infection, CAR/CXCR5 T cells accumulated in lymphoid follicles, came in direct contact with SIV-producing cells, and, in some instances, were associated with decreased SIV viral loads (VLs).^10^

One major barrier for HIV therapeutic studies is the availability of animal models to study HIV pathogenesis. While nonhuman primate (NHP) models infected with SIV mimic many pathologies caused by HIV, the use of NHP models is complicated by their genetic diversity, particularly at immunogenetic loci, which can determine their susceptibility to and maintenance of sustained infection.^11^ Such inconsistencies in NHP models can impose limitations on developing HIV therapeutics, as different types of NHPs infected with various strains of SIV can show as much as 30% spontaneous control of infection to low or undetectable levels in plasma,^12^^,^^13^ potentially complicating analyses of therapeutic efficacies. Additionally, most HIV immunotherapies cannot be used directly in SIV-infected NHP models of HIV because the HIV target molecules are not present in SIV. Given these limitations, various simian/HIV (SHIV) viruses have been investigated as models for HIV because they express HIV envelope (HIV-env).^14–20^ However, the inconsistency of viremia and the finding that many NHPs spontaneously control SHIV infections make these models problematic for testing the efficacy of HIV immunotherapies.^21–23^ Consequently, findings from SIV/SHIV studies in NHP models may not directly translate to HIV therapeutic testing.

Humanized mice are valuable in vivo models for studying HIV pathogenesis,^24–26^ HIV cellular immunotherapies,^27–30^ and HIV vaccines,^31^^,^^32^ as they are susceptible to HIV infection, sustain high VLs, show CD4^+^ T-cell depletion, and maintain HIV reservoirs.^33^ Importantly, products developed for treating HIV infections can be tested directly in HIV-infected humanized mice, without modification. The development and use of HLA-transgenic humanized mice improve reconstitution of functional human T and B cells after infusing HLA-matched human hematopoietic stem cells (hu-HSCs).^34–36^ Humanized (h) DRAGA mice have been engineered to express human HLA-A2.1 and HLA-DR0401 transgenes on a Rag1KO.IL2RγcKO.NOD (NRG) background.^37^ Human HLA expression in the thymus of these mice promotes the development of functional T and B cells from engrafted HLA-matched stem cells.^20^^,^^21^ hDRAGA mice develop diverse human cell types. In circulating blood, DRAGA mice reconstitute human T- and B-cell subsets,^37^^,^^38^ including populations of huCD4^+^CD25^+^FoxP3^+^ and huCD8^+^CD25^+^FoxP3^+^ regulatory T cells^38^ as well as CXCR5^hi^PD-1^hi^ germinal center T Follicular Helper cells.^39^ NK cells represent approximately 2% of hCD45^+^ cells in blood and 4% in spleen (Sofia Casares, personal communication). In addition, hDRAGA mice develop human epithelial and endothelial cells in lungs,^40^^,^^41^ human hepatocytes, Kupffer cells in the liver,^42^ and human microglia and human resident T cells in the brain.^43^ Human B cells in hDRAGA mice are capable of immunoglobulin class switching and elicit specific human cellular and antibody responses after vaccination.^37^^,^^38^^,^^44^^,^^45^ T cells in hDRAGA mice are proficient at secreting human IL-2, IFN-γ, TNF-α, IL-4,^37^ IL-13, IL-10, IL-17, MIP-1b, MCP-1, IL-18,^46^ and IL-21,^47^ as well as perforin and granzyme.^38^ T cells in hDRAGA mice also express numerous costimulatory molecules.^38^ Because hDRAGA mice develop diverse human cell types, they are a versatile model to study various human diseases including HIV^38–43^^,^^46^ and neurological conditions.^43^

Unlike most other humanized mice, hDRAGA mice develop follicle-like structures in lymph nodes and spleen,^39^ a critical feature of the model, as HIV replication is concentrated in lymphoid follicles.^48–52^ Importantly, HIV virions were shown to replicate in follicular-like CD20^high^ areas of secondary lymphoid tissues of HIV-infected hDRAGA mice,^39^ thus making this model particularly valuable to study HIV infections and pathogenesis.

Here, we evaluated the hDRAGA mouse model to test an HIV immunotherapy. We produced functional HIV-specific CAR/CXCR5 T cells from splenocytes of hDRAGA mice and infused them into HIV-infected hDRAGA mice. CAR/CXCR5 T cells accumulated in B-cell follicle–like structures (CD20^high^ areas) and were observed to be in direct contact with HIV RNA^+^ cells. The CAR/CXCR5 T cells administered intravenously were consistently detected in HIV-infected hDRAGA mice throughout the study. However, the CAR/CXCR5 T cells were insufficient to reduce HIV-1 VLs in plasma and were unable to prevent CD4^+^ T-cell loss postinfection, likely because the infused CD4^+^ CAR/CXCR5^+^ T cells and nontransduced cells in the product were susceptible to HIV infection. However, these studies show that HIV infection was treatment responsive in the DRAGA mice; all mice treated with ART showed complete suppression of viral replication. Our findings support the use of the hDRAGA mice as an appropriate model to test cellular immunotherapies.

## Materials and methods

### hDRAGA mice and ethical considerations

All animal procedures reported herein were conducted under Institutional Animal Care and Use Committee protocols approved by the Walter Reed Army Institute of Research/Naval Medical Research Command (WRAIR/NMRC) (22-28-AVAT) and the University of Minnesota (UMN) (2011-38633A) in compliance with the Animal Welfare Act and by the principles outlined in the “Guide for the Care and Use of Laboratory Animals,” Institute of Laboratory Animals Resources, National Research Council, National Academy Press, 2011.

DRAGA mice were bred and humanized at the Veterinary Service Program at WRAIR/NMRC. De-identified umbilical cord blood positive for HLA-A2.1 and HLA-DR0401 was commercially procured through the New York Blood Center (Long Island City, NY, United States).^53^ In brief, the mice were irradiated (350 rads) and injected intravenously with CD3^+^ T-cell–depleted cord blood (EasySep Human CD3 Positive Selection Kit, 18051, Stem Cell Technologies) containing approximately 10^5^ human CD34^+^ HSCs as determined by FACS using a mouse anti-human CD34 antibody (BD Biosciences, 550761). CD3 depletion of human T cells was required to avoid lethal (acute) graft-versus-host reaction. In this study, the mice were infused with hu-HSCs from one human cord blood donor (HLA haplotype A02:01A11:01B15:01B52:01DR04:01DR15:02), and the percentages of human T cells (CD3^+^, CD3^+^CD4^+^, and CD3^+^CD8^+^) and B cells (CD19^+^) were examined at WRAIR/NMRC at 18 wk posthumanization. The procedures for assessing percentages of human T and B cells by FACS on the mononuclear FSC/SSC gate have been described previously.^38^ Following the infusion, the hDRAGA mice were transferred to UMN. Prior to HIV infection, the hDRAGA mice were reevaluated for human reconstitution status by assessing the percentages of human CD45^+^ and human T cells in peripheral blood by flow cytometry.

### Animal study experimental design, HIV-1 infection of hDRAGA mice, and ART

Fourteen female hDRAGA mice (designated as DRAGA no. 1 through 14) were housed in UMN animal facilities. Housing conditions were temperature-controlled (22 °C ± 1 °C) on a light/dark cycle of 14 h/10 h with ad libitum access to irradiated food and acidified water (pH 2.9 ± 0.2). In this study, the mice were allocated into 2 groups: (1) control animals (HIV-infected only; n = 6) and (2) treated animals (HIV-infected, CAR/CXCR5-treated; n = 6). The remaining 2 animals without HIV infection were used for CAR/CXCR5 T-cell production. The hDRAGA mice were infected with HIV-1_Ba-L_ as described previously.^26^^,^^54^ Twelve hDRAGA mice were pretreated subcutaneously with 2.5 mg per 50 μL of medroxyprogesterone acetate (DMPA) (Mylan Institutional LLC) 7 d prior to intravaginal infection with 10,000 tissue culture infectious dose 50 (TCID_50_) of HIV-1_Ba-L_ in 20 μL PBS. HIV-1_Ba-L_ viruses (ARP-510) were obtained from the National Institutes of Health (NIH) HIV reagent program.^47^ The hDRAGA mice were checked biweekly for HIV-1 levels in plasma. The hDRAGA mice testing negative for HIV-1 at 2 wk postinoculation were reinfected with the same infectious dose of HIV and rechecked for HIV-1 levels in plasma. After the plasma HIV-1 VLs had plateaued, the transduced CAR/CXCR5 T-cell products were infused into the treated animals. The hDRAGA mice were sacrificed at 6, 14, and 28 d postinfusion (DPI).

In the ART study, 14 female and 8 male mice were housed in conditions described above. These mice were infected intraperitoneally with 20,000 TCID_50_ of HIV-1_Ba-L_ in 200 µL PBS. Viral loads and weight were monitored weekly, and CD4 counts were monitored biweekly. Four weeks after infection, the mice were switched from standard feed to 1/2″ pellets of irradiated Teklad chow 2020X containing 1,500 mg emtricitabine, 1,560 mg tenofovir disoproxil fumarate, and 600 mg raltegravir per kilogram (Research Diets, New Brunswick, NJ, United States) as previously described.^24^ hDRAGA mice were free fed, and chow levels were monitored daily to ensure adequate consumption. Chow was refreshed weekly.

### Production of CAR/CXCR5 T cells from the spleens of hDRAGA mice

Spleens from 2 non-HIV-infected hDRAGA mice were collected and disaggregated. Disaggregated cells from one of these mice were used to produce engineered CAR/CXCR5 T cells containing a bispecific CD4-MBL CAR and encoding CXCR5 by adapting our previously published methods.^9–11^^,^^55^ The transduction efficacy of CAR/CXCR5 T cells was evaluated by assessing the co-expression of MBL and CXCL5 in live CD3^+^ cells using flow cytometry. The gating strategy included the following steps: identifying lymphocytes (SSC-A vs FSC-A), singlets (FSC-H vs FSC-Width), viable cells (SSC-A and Live/Dead NIR), mouse hematopoietic cells (SSC-A and mCD45^+^), human hematopoietic cells (SSC-A and hCD45^+^), human T cells (SSC-A and CD3^+^), and finally, CAR/CXCR5 T cells (MBL^+^ and CXCR5^+^).

### Intracellular cytokine staining

Antigen-specific production of TNF-α and IFN-γ and surface expression of CD107a were assessed by co-culturing CAR/CXCR5 cells with HIV-env K562 cells (kindly provided by Dr James Riley, University of Pennsylvania) or wild-type (WT) K562 cells (ATCC) at an E:T ratio of 3:1. Anti-human CD107a was added 1 h prior to incubation with brefeldin A and monensin, and cells were then incubated for 3 h at 37 °C. Intracellular cytokine staining was performed for TNF-α and IFN-γ prior to surface staining for MBL, CXCR5, and CD3. Samples were acquired on a CytoFLEX (Beckman) and analyzed via FlowJo version 10.2 software (Becton Dickinson). Cells were gated on lymphocytes, single cells, live cells, CD3^+^MBL^+^CXCR5^+^, and then TNF-α^+^, IFN-γ^+^, or CD107a^+^.

### Cell cytotoxicity assays

The DELFIA EuTDA assay (Revvity, AD0116) was used to assess cell cytotoxicity per the manufacturer’s instructions. In brief, HIV-env K562 and WT K562 cells were labeled with BATDA for 30 min at 37 °C and subsequently washed with PBS. CAR/CXCR5 T cells or untransduced (UTD) T cells were plated with HIV-env K562 or WT K562 cells at 1:1, 5:1, 10:1, and 20:1 E:T ratios. After 2 h of incubation, 20 μL of supernatant was added to 200 μL of Europium solution and transferred to a flat-bottom plate, and time-resolved fluorescence was measured using a Synergy 2 (Biotek). Spontaneous and maximum release wells were prepared by adding BATDA-loaded K562 cells to a well containing medium alone or medium with 10% lysis buffer, respectively. Specific release was defined as (experimental release–spontaneous release)/(maximum release–spontaneous release).

### Migration assay

Migration of CAR/CXCR5 T cells toward specific human cytokines was conducted as previously described.^9^^,^^55^ In brief, 1 × 10^6^ CAR/CXCR5 T cells and UTD T cells (in 100 µL of X-VIVO 15 with 0.1% BSA) was placed in the upper chamber of a Transwell plate (Corning Costar, CLS3401-48EA) with a 5.0-µm membrane. The lower chamber was filled with 600 µL X-VIVO with 0.1% BSA alone or containing human CXCL13 (2.5 µg/mL) (Peprotech, 300-47-5UG). After a 4-h incubation at 37 °C, the cells were collected and counted using a CytoFLEX flow cytometer (Beckman Coulter). AccuCheck counting beads (Invitrogen, PCB100) were added to each sample to ensure accurate cell counts. Specific migration was defined as (cells that migrated to CXCL13^–^ cells that migrated to medium alone)/input cells.

### Infusion of transduced CAR/CXCR5 cell products

Prior to CAR/CXCR5 T-cell infusion, the control and treated animals were allocated based on age, weight, peak of plasma HIV-1 RNA VLs, levels of plasma HIV RNA VLs before the infusion of transduced cell products, CD4^+^/CD8^+^ ratios, and CD4^+^ T-cell count (Fig. S1). The treated animals were infused intravenously with transduced cell products (2 × 10^5^ cells/g body weight). The animals were monitored twice daily for any signs of pain, illness, and stress by observing appetite, stool, behavior, and physical condition in response to the infused CAR/CXCR5 T cells.

### Blood and tissue collections

Peripheral blood samples were collected in blood collection tubes containing EDTA anticoagulant (Sarstedt AG & Co., 41.1395.105) pre- and postinfection, the day of CAR/CXCR5 T-cell infusion, and at 6, 14, and 28 DPI. Peripheral blood (up to 100 μL) was centrifuged at 1200 × *g* for 10 min, and plasma was collected as described previously.^26^^,^^39^ The remaining peripheral blood (50 μL) was processed for cell phenotypes and CD4^+^ T-cell count.

Spleens and lymph nodes were disaggregated using a 70-µm nylon cell strainer (Corning, 352350). For the spleens, red blood cells were lysed with ACK lysing buffer (Gibco, A1049201) according to the manufacturer’s instructions. Single-cell suspensions were stained for analysis using flow cytometry.

### Quantification of plasma HIV-1 viral loads

Cell-free HIV-1 RNA was isolated from 50 μL plasma using a QIAamp Viral RNA Mini Kit (Qiagen, 52906) according to the manufacturer’s instructions. HIV-1 quantitation real-time PCR was performed using an HIV Quantitative TaqMan RT-PCR Detection Kit (Norgen Biotek Corp.) and a CFX96 Real-time PCR System (C100 Touch). Data were collected and analyzed with the CFX Manager 3.1 software (Bio-Rad).

### Quantitation of CD4^+^ T-cell count in peripheral blood

Quantification of CD4^+^ T-cell numbers in peripheral blood was conducted using flow cytometric frequency gates, as described previously.^56^ In brief, peripheral blood samples (50 μL) were treated with ACK lysing buffer (Gibco, A1049201) to lyse red blood cells. After centrifugation, cells were resuspended in 200 μL 1× PBS; 50 μL of the cell suspension was mixed with the AccuCheck counting beads (Thermo Fisher Scientific, PCB100) and counted on a CytoFLEX flow cytometer (Beckman Coulter), while the remaining suspended cells were stained for cell phenotyping. The data were analyzed with FlowJo software version 10 (BD Life Sciences). The CD4^+^ T-cell count was analyzed based on the percentage of live hCD3^+^MBL^–^CD4^+^ in lymphocytes compared to the number of beads and lymphocytes.

### Flow cytometry and antibodies

The resuspended cells were stained with anti-mouse antibodies mCD45 (BioLegend, 30-F11, 103149) and the following anti-human antibodies: hCD45 (BD Pharmingen, HI30, 557748), CD4 (BD Pharmingen, M-T477, 556615), CD3 (BD Pharmingen, P34.2, 557917), Live/Dead NIR (Invitrogen, L34976A), CD185 (CXCR5) (MU5UBEE, 12-9185-42), MBL (Invitrogen, 3E7, custom-made and labeled with Alexa Fluor 647, Invitrogen, A20186); CD45RA (BD Pharmigen, 5H9), CD45RO (BioLegend, UCHL1), CD27 (BD Pharmigen, M-T271), CCR7 (BD Pharmigen, 150503), and CD8 (BioLegend, SK1, 344731). Flow cytometric data were acquired on a Beckman Coulter CytoFLEX and analyzed with FlowJo software version 10 (BD Life Sciences).

### RNAscope in situ hybridization combined with immunofluorescence

In this study, we selected 3 slides from one treated hDRAGA at 6 DPI and one slide from one control hDRAGA at 6 DPI. RNAscope multiplex fluorescent kit V2 (Advanced Cell Diagnostics, UM 323100) with the opal fluorophores system was used to simultaneously detect HIV viral RNA (vRNA)^+^ and transduced CAR/CXCR5 cells according to the manufacturer’s instructions and as described previously.^10^^,^^57^ For duplex detection of HIV vRNA^+^ and transduced CAR/CXCR5 cells, the sectioned slides were incubated overnight at 40 °C with the C2 probe (Advanced Cell Diagnostics, V-HIV-1-Clade B 416111-C2) to detect HIV vRNA^+^ cells and a customized C1 probe to detect the CAR/CXCR5 cells. The sections were then washed with 0.5× RNAscope wash buffer and incubated with amplification reagents^1–3^ according to the manufacturer’s instructions. Opal 570 (Akoya Bioscience, FP1488001KT) and Opal 650 (Akoya Bioscience, FP1496001KT) were used for the C1 and C2 probes, respectively.

For immunofluorescence staining, the sections were conducted as described previously^10^ with some modifications. In brief, the sections were stripped, washed, blocked, and incubated overnight at 4 °C with 0.51 μg/mL rabbit anti-human CD20 antibodies (Abcam, EP459Y, ab78237) in 10% normal goat serum (NGS)-TBS-1% BSA. Then, sections were washed and incubated with 7.5 μg/mL secondary Alexa Fluor 488–conjugated polyclonal goat anti-rabbit IgG antibodies (Jackson ImmunoResearch, 111-545-003) in 10% NGS-TBS-1% BSA, counterstained with 1 μg/mL DAPI solution (Thermo Scientific, 2247), and mounted in Prolong Gold antifade reagent (Invitrogen, P36934). The stained tissue sections were imaged with a Leica TCS SPE DM6000 confocal microscope.

### Statistical analysis

All statistical analyses were performed using GraphPad Prism 10 software. Data are represented as mean ± SD, median, or geometric mean with 95% confidence interval (CI). Groups were compared using the Mann–Whitney test. A *P* value of <0.05 was considered significant.

## Results

### hDRAGA mice reconstituted with human immune cells and infected with HIV showed sustained high HIV plasma viral loads

Fourteen female hDRAGA mice were assessed for human reconstitution at 18 wk post–hu-HSC infusion at WRAIR/NMRC before the animals were transferred to UMN. The human immune cell reconstitution status in peripheral blood is shown in Table 1.

**Table 1.** Reconstitution of human immune cells in hDRAGA mice from WRAIR/NMRC.

Mouse #	Frequency (%) of lymphocytes in peripheral blood			
	CD19^+^	CD3^+^	CD3^+^CD4^+^	CD3^+^CD8^+^
**DRAGA-1**	57.7	8.5	7.9	0.6
**DRAGA-2**	52.6	18	15.3	2.7
**DRAGA-3**	75.8	10.8	8.7	2.1
**DRAGA-4**	46.3	20.6	18.9	1.7
**DRAGA-5**	64.2	13.3	11.8	1.5
**DRAGA-6**	66.1	12.1	11.3	0.8
**DRAGA-7**	61.7	12.2	10.7	1.5
**DRAGA-8**	67.8	9.8	7.7	2.1
**DRAGA-9**	69.0	9.4	7.4	2.0
**DRAGA-10**	66.7	11.5	10.4	1.1
**DRAGA-11**	61.0	9.7	9.1	0.6
**DRAGA-12**	50.0	13.0	12.1	0.9
**DRAGA-13**	54.6	10.6	9.7	0.9
**DRAGA-14**	52.9	16.0	14.9	1.1

**Figure 1.**
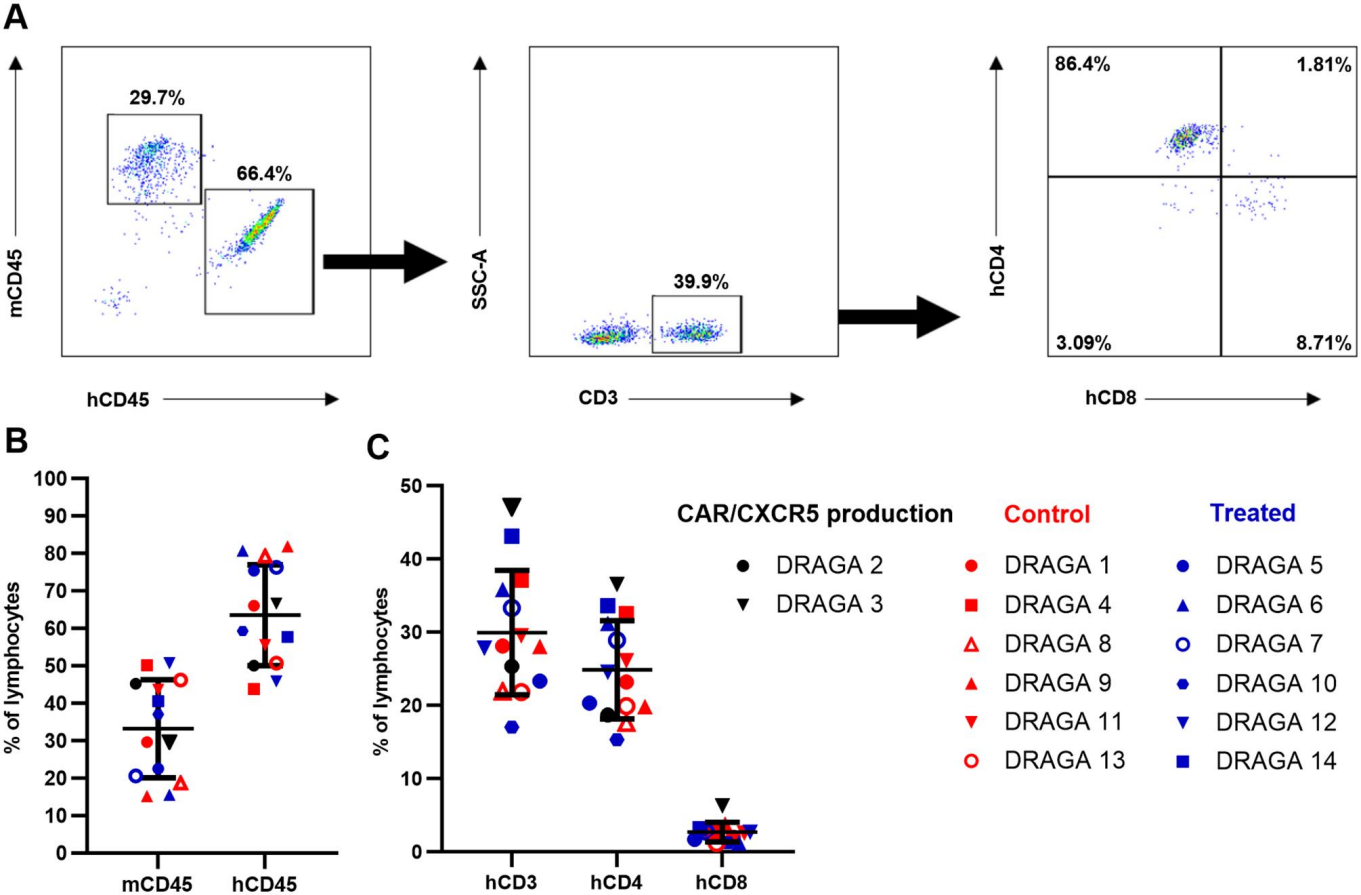
Human T-cell reconstitution in peripheral blood from hDRAGA mice. Peripheral blood samples from all hDRAGA mice (n = 14) were examined by flow cytometry for the level of human immune cell reconstitution prior to infection with HIV-1. (A) An example of the gating strategy for quantifying human immune cell reconstitution in an hDRAGA mouse. (B) Percentages of mouse CD45^+^ and human CD45^+^ cells in lymphocytes. (C) Percentages of human CD3^+^, CD4^+^, and CD8^+^ T cells. Data are mean ± SD.

We reexamined the reconstitution of human immune cells in all hDRAGA mice (n = 14) at 25 wk after the hu-HSC infusion (Fig. 1). The 14 hDRAGA mice showed high frequencies of human hematopoietic-derived cells (Fig. 1A), identified by human CD45^+^ cells (63.51 ± 13.49%) in their peripheral blood samples (Fig. 1B). The hDRAGA mice showed a reconstituted profile of human CD3^+^ T cells (29.94 ± 8.51%), human CD4^+^ T cells (24.86 ± 6.7%), and human CD8^+^ T cells (2.69 ± 1.33%) (Fig. 1C).

**Figure 2.**
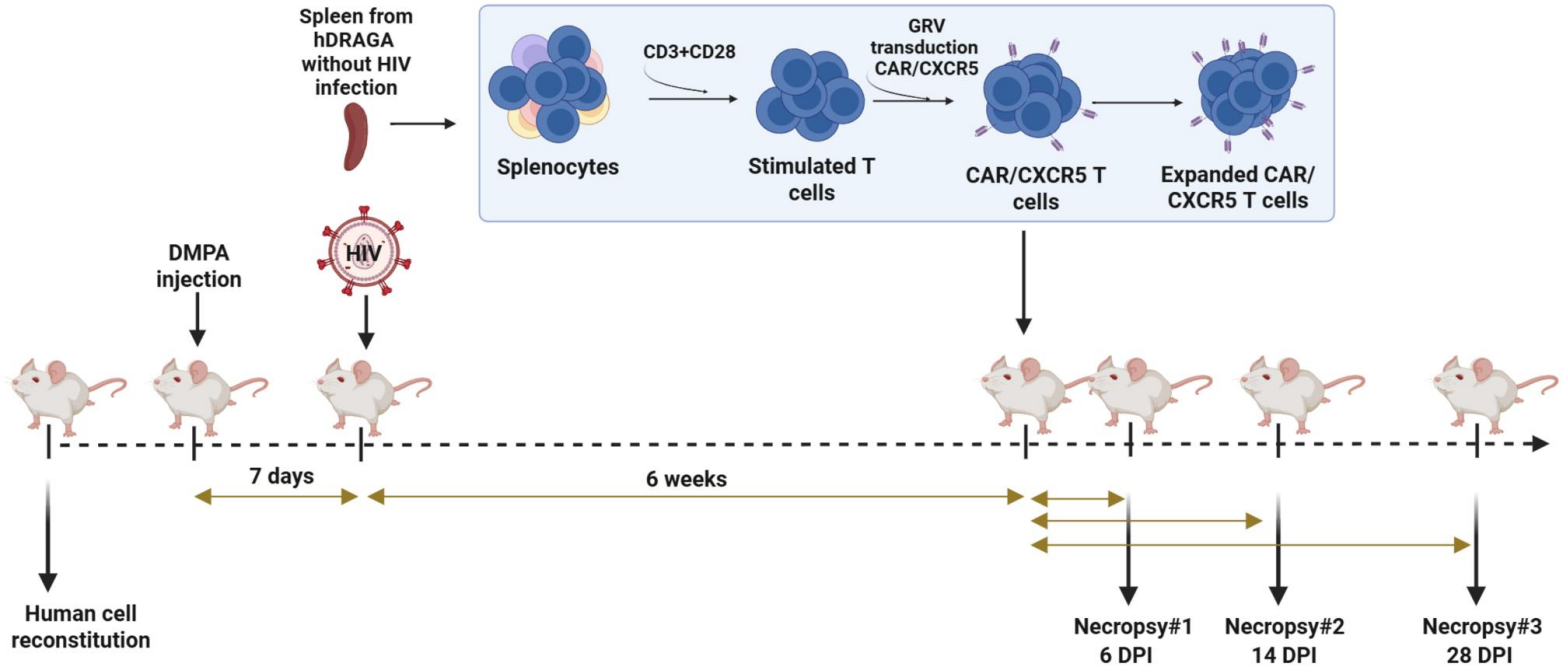
Timeline of CAR/CXCR5 T-cell immunotherapy in hDRAGA mice. Animals (n = 14) were reevaluated for human cell levels, treated with DMPA (n = 12), and 7 d later infected with HIV. Splenocytes from 1 of 2 uninfected hDRAGA mice were used to engineer CAR/CXCR5 T cells. Six weeks after HIV infection, animals were infused with CAR/CXCR5 T-cell products or were untreated, with subsets of animals sacrificed at 6, 14, and 28 DPI.

Six of the 12 HIV-infected hDRAGA mice were treated with CAR/CXCR5 T cells and subsequently sacrificed to evaluate the efficacy and persistence of the CAR/CXCR5 T cells, as outlined in the experimental timeline for CAR/CXCR5 immunotherapy shown in Fig. 2.

**Figure 3.**
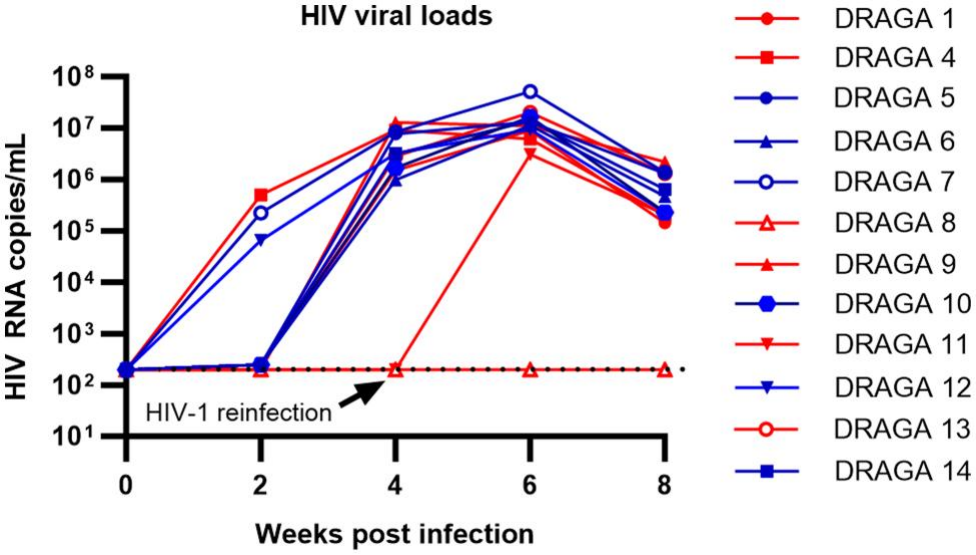
HIV-1 RNA plasma VLs prior to CAR/CXCR5 T-cell infusion. Peripheral blood samples were collected biweekly from hDRAGA via facial vein. DRAGA 8 and 11 were reinfected with HIV-1 at 4 wk postinfection. The level of HIV-1 RNA in plasma was quantified using qRT-PCR for CAR/CXCR5 T-cell–treated (blue) and control (red) animals The threshold of HIV RNA detection was 200 copies/mL (dashed line).

The hDRAGA mice were monitored biweekly for plasma HIV-1 VLs after day 1 of HIV-1_Ba-L_ inoculation, as assessed by quantitative RT-PCR (qRT-PCR) assays. Overall, 11 of the 12 (91.67%) hDRAGA mice were HIV-infected. Ten hDRAGA mice were positive for HIV-1 in their plasma by 4 wk postinfection with a single HIV-1_Ba-L_ infection (Fig. 3). Two hDRAGA mice (DRAGA 8 and 11) were negative for HIV-1 infection at 4 wk and were reinfected with HIV-1_Ba-L_ (Fig. 3). Two weeks later, DRAGA 11 showed HIV infection, while DRAGA 8 remained below the detection limit, resulting in the exclusion of this animal in this study. The time of peak plasma VL varied among the HIV-infected mice (Fig. 3).

Prior to infusion with CAR/CXCR5 T cells, 11 HIV-infected hDRAGA mice were allocated based on the criteria described in Fig. S1, which included age, the peak of plasma HIV RNA VLs, levels of plasma HIV RNA VLs prior to infusion of transduced T-cell products, weight, human CD4^+^/CD8^+^ T-cell ratios, and human CD4^+^ T-cell counts. The average CD4^+^ T-cell counts in the control animals (geometric mean: 666 [95% CI: 259–1712] cells/μL) were lower than those in the treated animals (geometric mean: 1590 [95% CI: 630–4009] cells/μL), but this difference was not significant (*P* = 0.093).

### Functional CAR/CXCR5 T cells were produced from hDRAGA splenocytes

**Figure 4.**
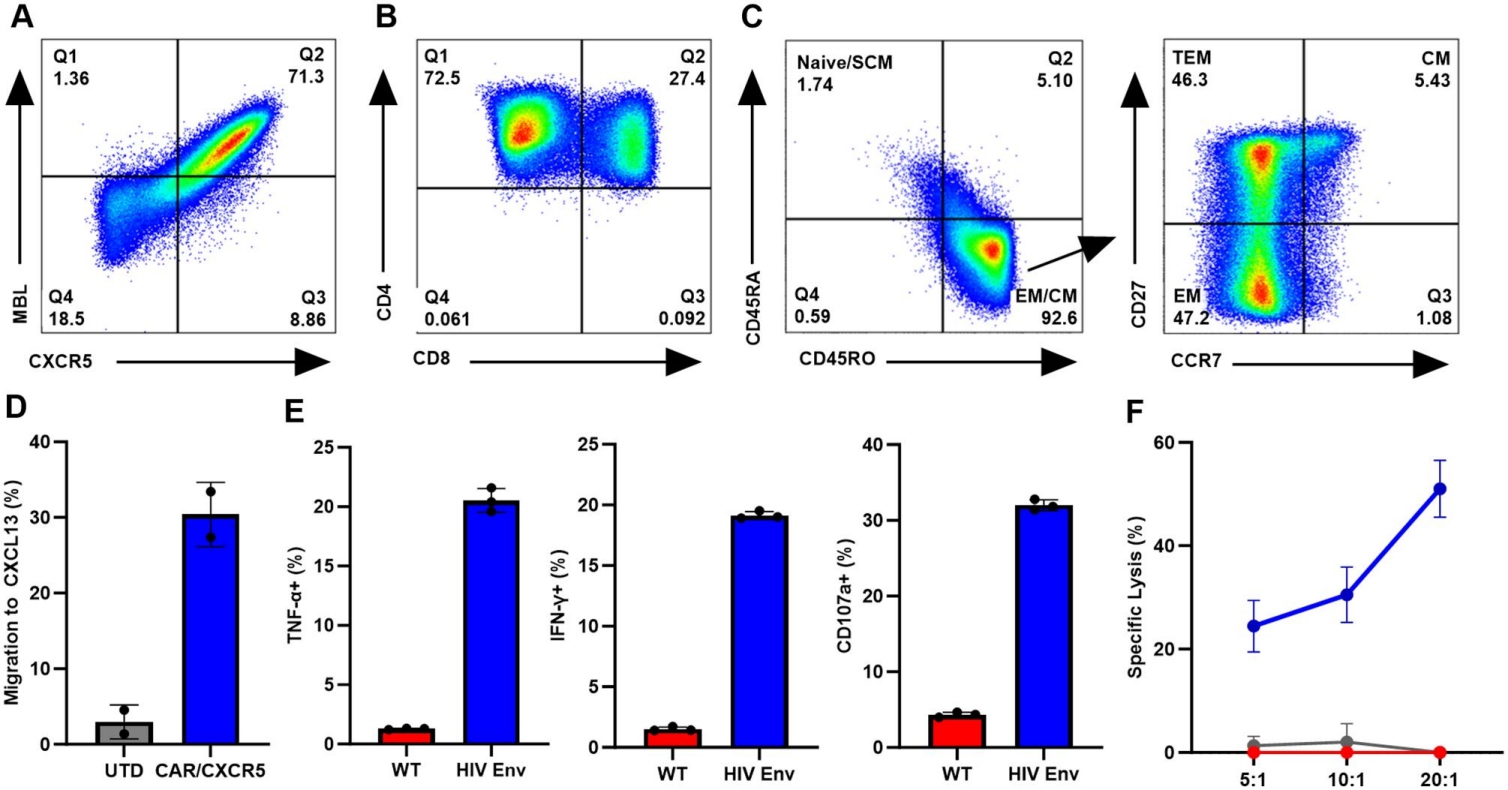
Characterization of transduced CAR/CXCR5 T cells produced from splenocytes of an hDRAGA mouse. (A) CAR/CXCR5 transduced cells from one DRAGA spleen were identified by expression of MBL and CXCR5. A gate was set on the control UTD cells (not shown). (B) CD4^+^ and CD8^+^ expression within MBL^+^CXCR5^+^ T cells. (C) Memory populations were gated within MBL^+^CXCR5^+^ transduced T cells and identified as naive or stem cell memory (SCM) (CD45RA^+^, CD45RO^–^), central memory (CM) (CCR7^+^, CD27^+^), transitional effector memory (TEM) (CCR7^–^, CD27^+^), or effector memory (EM) (CCR7^–^, CD27^+^). Gates were set on lymphocytes, singlets, and live human CD3^+^ T cells for (A), and additionally on MBL^+^CXCR5^+^ cells for (B) and (C). (D) Migration to CXCL13 by control UTD (gray) or CAR/CXCR5 T cells (blue) was measured in a transwell migration assay. (E) CAR/CXCR5 T cells were co-cultured with either WT (red) or HIV-env–expressing K562 cells (blue) at a 1:3 E:T ratio and the expression of TNF-α, IFN-γ, and CD107a were measured. Gates were set on lymphocytes, singlets, and live human CD3^+^ T cells for (A), and on MBL^+^CXCR5^+^ cells for (D). (F) A DELFIA cytotoxicity assay was performed at 5:1, 10:1, and 20:1 E:T ratios with CAR/CXCR5 T cells plus HIV-env K562 cells (blue), CAR/CXCR5 T cells plus WT K562 cells (red), or UTD T cells plus HIV-env K562 cells (gray).

Disaggregated splenocytes from a non-HIV-infected hDRAGA mouse were used to produce CAR/CXCR5 T cells. Flow cytometric analysis showed that approximately 72% of CD3^+^ T cells co-expressed MBL (a portion of the extracellular domain of this CAR molecule) and CXCR5 molecules (Fig. 4A). Of the transduced cells expressing CAR/CXCR5, 72.5% were CD4^+^CD8^–^ and 27.5% were CD4^+^CD8^+^ T cells (Fig. 4B). Note that the CD4 antibodies also detect the CD4-MBL-CAR molecule and, as such, the CD8^+^CAR/CXCR5 T cells appear as both CD4^+^ and CD8^+^. CD4^+^ cells are included in the CAR/CXCR5 T-cell product as CD4^+^ CAR T cells exhibit beneficial cytotoxic activity in addition to their “helper” functions.^58–61^ The CAR/CXCR5 T cells were found to be approximately 1.7% naive and stem cell memory, 5.4% central memory, 46.3% transitional effector memory (T_TEM_), and 47.2% effector memory (T_EM_) (Fig. 4C).

The functionality of CAR/CXCR5 T cells was determined through in vitro assays. CAR/CXCR5 T cells showed almost a 6-fold increase in migration to CXCL13 when compared to UTD cells (Fig. 4D), demonstrating the functionality of the CXCR5 molecule. Additionally, the CAR cells demonstrated specific activity against HIV-env–expressing target cells. CAR/CXCR5 T cells released elevated levels of TNF-α, IFN-γ, and CD107a when co-cultured with HIV-env K562 cells compared to co-culture with WT K562 cells (Fig. 4E). CAR/CXCR5 T cells specifically killed HIV-env K562s at high rates compared to UTD cells, and showed low off-target killing of WT K562s (Fig. 4F). Taken together, these data demonstrate that highly functional CAR/CXCR5 T cells can be generated from DRAGA splenocytes.

### CAR/CXCR5 T cells were safe when administered to HIV-infected hDRAGA mice

We evaluated the safety of donor-matched CAR/CXCR5 T-cell immunotherapy treatment in HIV-infected hDRAGA mice, compared to control mice after the infusion of the transduced CAR/CXCR5 T-cell product. Our previous study in NHPs showed that infusion of CAR/CXCR5 T cells at a dosage of 2 × 10^8^ cells/kg body weight (equivalent to 2 × 10^5^ cells/g in mice) was safe and effective in reducing SIV VLs in SIV-infected ART-treated and -released rhesus macaques.^10^ This dose led to the highest effector CAR/CXCR5 T cells to target SIV vRNA^+^ cell ratios in spleen and lymphoid follicles. Thus, the CAR/CXCR5 T cells were infused at a dose of 2 × 10^5^ cells/g in this study. The CAR/CXCR5 T-cell–treated mice exhibited no noticeable adverse effects after receiving the immunotherapeutic cells, and their weights were unaffected by the immunotherapeutic infusion. Necropsy showed no abnormalities of internal organs in treated animals beyond those typical in HIV-infected hDRAGA animals.

### CAR/CXCR5 T cells persisted in peripheral blood and secondary lymphoid tissues

**Figure 5.**
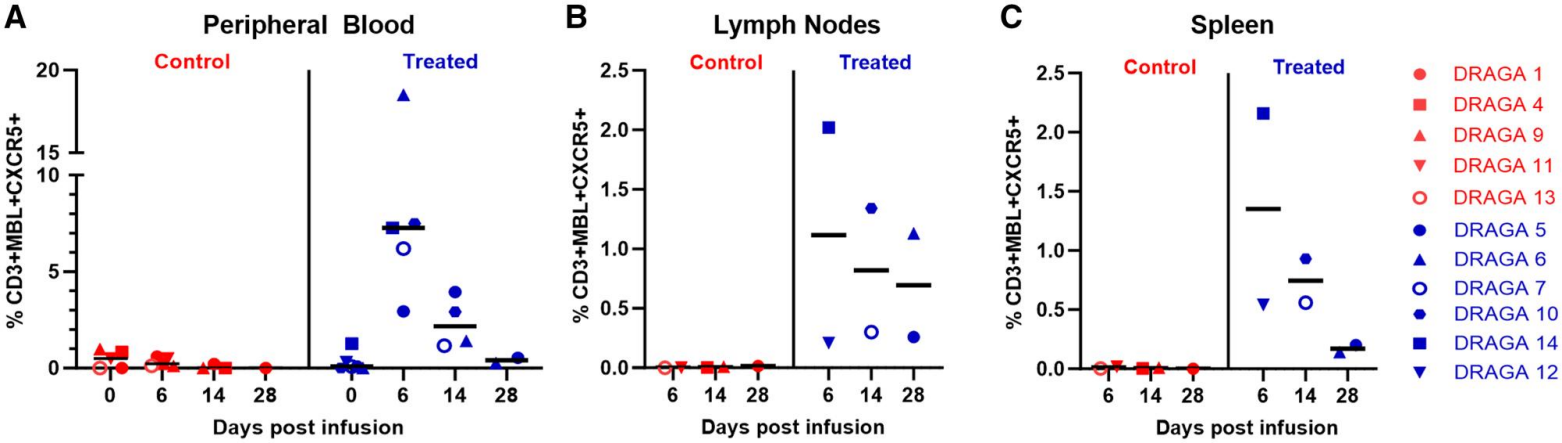
Persistence of CAR/CXCR5 T cells in HIV-infected hDRAGA mice postinfusion. CAR/CXCR5 T cells were enumerated in peripheral blood (A), lymph nodes (B), and spleen (C) of hDRAGA mice at 6, 14, and 28 DPI. Each graph shows the percentages of MBL^+^ and CXCR5^+^ cells (CAR^+^ T cells) within the live CD3^+^ T-cell population. The bar represents median percentages.

After infusion, CAR/CXCR5 T cells were detected in the peripheral blood (Fig. 5A), lymph nodes (Fig. 5B), and spleens (Fig. 5C) of the treated mice through the end of the study at 28 DPI. In all of the disaggregated cell samples, the number of CAR/CXCR5 T cells was highest at 6 DPI and gradually decreased over time.

### CAR/CXCR5 T cells colocalized with HIV vRNA^+^ cells within CD20^high^ areas

**Figure 6.**
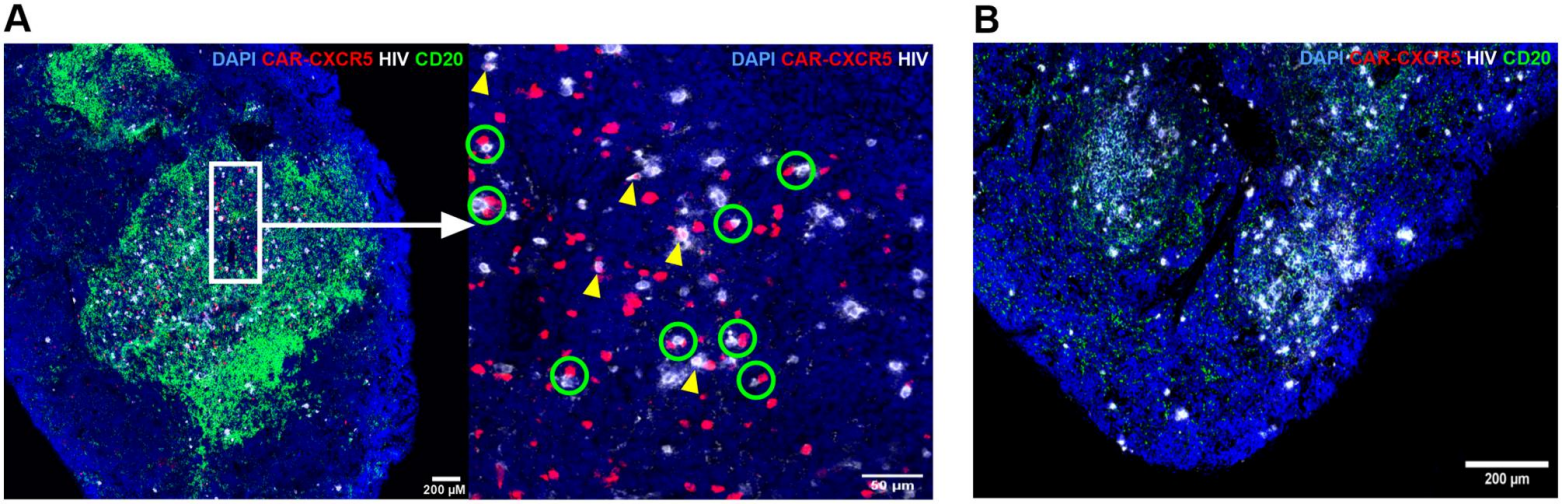
Localization of CAR/CXCR5 T cells and HIV vRNA^+^ cells. RNAscope in situ hybridization combined with immunohistochemistry showed that CAR/CXCR5 T cells (red) and HIV vRNA^+^ cells (white) localize in follicle-like structures of the spleen of a representative CAR/CXCR5 T-cell–treated hDRAGA mouse and UTD control HIV-infected hDRAGA mouse at 6 DPI. (A) In a spleen from a representative treated hDRAGA mouse (left), the enlarged image (right) shows CAR/CXCR5 T cells in direct contact with HIV vRNA^+^ cells, indicated in the green circles. Several CAR/CXCR5 T cells were HIV vRNA^+^ (yellow arrowheads). (B) RNAscope analysis of the spleen from a representative control HIV-infected hDRAGA mouse detected HIV vRNA^+^ cells (white) but not CAR/CXCR5 T cells (red). B-cell follicle–like structures were identified using anti-human CD20 antibodies (green), and nuclei were stained with DAPI (blue). The confocal image was collected with a 20× magnification (Numerical aperture = 0.4).

We investigated the localization of the CAR/CXCR5 T cells and HIV vRNA^+^ cells in a spleen of an infused hDRAGA mouse using RNAscope combined with immunohistochemistry (Fig. 6A). At 6 DPI, the spleen from a CAR/CXCR5 T-cell–treated hDRAGA mouse (DRAGA no. 14) showed abundant accumulation of both HIV vRNA^+^ cells and CAR/CXCR5 T cells within the CD20^high^ B-cell follicle–like structures (Fig. 6A). Importantly, many CAR/CXCR5 T cells were found to be in direct contact with HIV vRNA^+^ cells (Fig. 6A). However, as seen in Fig. 6A, many CAR/CXCR5 T cells were also HIV vRNA^+^. In a control HIV-infected hDRAGA mouse, HIV vRNA^+^ cells were detected and accumulated within the CD20^high^ B-cell–containing follicle-like structures, and no CAR probe signal was detected (Fig. 6B).

### Transduced CAR/CXCR5 T cells were insufficient to reduce plasma HIV-1 viral loads during active HIV infection

**Figure 7.**
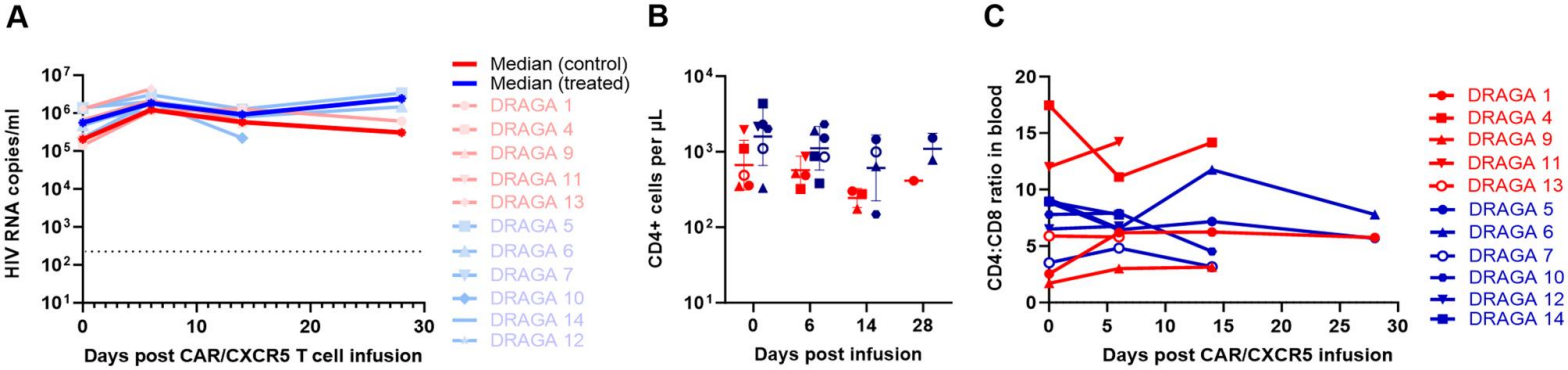
Effect of CAR/CXCR5 T-cell infusion on plasma HIV VLs and CD4^+^ T-cell count. (A) The HIV VLs were measured in the CAR/CXCR5 T-cell–treated and untreated control mice at 0, 6, 14, and 28 DPI using qRT-PCR. Differences were evaluated between control and treated animals using Mann–Whitney test (0 DPI, *P* = 0.833; 6 DPI, *P* = 0.833; 14 DPI, *P* > 0.99). The threshold of HIV RNA detection was 200 copies/mL (dashed line). (B) CD4^+^ T-cell counts were conducted for the CAR/CXCR5 T-cell–treated and UTD control mice at 0, 6, 14, and 28 DPI. Differences were evaluated between control and treated animals using Mann–Whitney test (0 DPI, *P* = 0.126; 6 DPI, *P* = 0.310; 14 DPI, *P* = 0.400). (C) CD4^+^/CD8^+^ ratios post–CAR/CXCR5 T-cell infusion in the PBMCs. Red dots and blue dots represent the control and treated animals, respectively. The red and blue lines (A) represent the median VLs in the control and treated animals, respectively. Bar and error (B) indicate mean ± SD. The red and blue lines with symbols (C) represent individual animals from each group.

The difference in plasma HIV VLs between treated and control animals was not statistically significant (*P* > 0.05 at all time points) (Fig. 7A and Table S1). The mean of CD4^+^ T-cell counts declined slightly in both control and treated animals, but there was no statistically significant difference between the 2 groups at each time point (*P* > 0.05 at all time points) (Fig. 7B). Likewise, infusion of the CAR/CXCR5 T-cell product did not impact the CD4^+^/CD8^+^ ratios (Fig. 7C).

### ART led to viral suppression in HIV-infected hDRAGA mice

**Figure 8.**
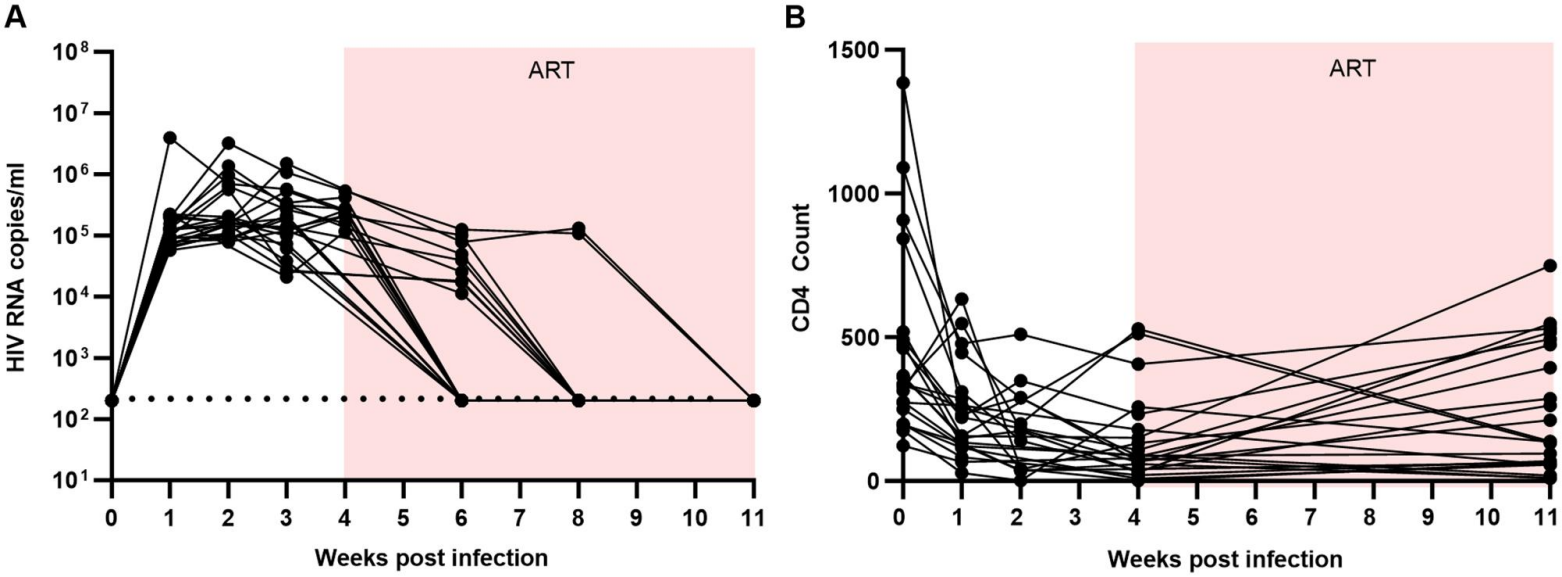
HIV-1 RNA plasma VLs and CD4 counts before and during ART. Twenty-two hDRAGA mice were infected with HIV and then peripheral blood samples were collected weekly until ART onset and then biweekly via facial vein. Mice were put on ART-containing chow 4 wk postinfection (pink box). (A) The level of HIV-1 RNA in plasma was quantified using qRT-PCR. The threshold of HIV RNA detection was 200 copies/mL (dashed line). (B) CD4^+^ T-cell counts were detected from peripheral blood samples and quantified using flow cytometry.

As CAR/CXCR5 T cells were unable to suppress HIV during active infection, we wanted to confirm that HIV-infected hDRAGA mice were capable of being virally suppressed. A total of 22 hDRAGA mice (8 male and 14 female) were intraperitoneally infected with HIV-1_Ba-L_. This method yielded a 100% first-time infection rate compared to intravaginal infection, and no reinfection was required. After 4 wk, animals were given ART-containing chow instead of regular feed. Viral loads were undetectable in 13 of 22 hDRAGA by 2 wk post-ART (6 wk postinfection), increasing to 20 of 22 by 4 wk post-ART (8 wk postinfection) (Fig. 8A). All animals had undetectable VLs by 7 wk post-ART (11 wk postinfection) (Fig. 8A). Additionally, CD4 counts increased in 15 of 22 animals during ART (Fig. 8B). These data indicate that hDRAGA are a suitable model for studying HIV therapeutics as they respond to ART in a similar to humans.

## Discussion

Overall, the results from these studies demonstrate that hDRAGA mice are a valuable model to study HIV immunotherapies. Indeed, in this model we were able to produce immunotherapeutic cells from the spleen of an hDRAGA mouse, track immunotherapeutic cells in blood and tissues, monitor VL in blood, track vRNA^+^ cells in tissues, and perform in situ analyses in spleen tissues to localize and quantify HIV vRNA^+^ cells and CAR/CXCR5 T cells simultaneously. We found that treating HIV-infected hDRAGA mice with a high dose of CAR/CXCR5 T cells during chronic infection was safe. We also found that HIV infection in hDRAGA mice is readily suppressed to undetectable levels using ART.

We found that hDRAGA mice had high levels of reconstituted human immune cells, were susceptible to HIV infection, sustained high HIV-1 VLs, and found that the hDRAGA mice developed B-cell–containing follicle-like structures in their secondary lymphoid tissues and that HIV vRNA^+^ cells accumulated in these structures, supporting the findings by Ollerton et al.^39^

Humanized DRAGA splenocyte–derived CAR/CXCR5 T cells were functional and specifically killed secreted cytokines in response to HIV-env–expressing target cells, and migrated to CXCL13 signaling in vitro. In vivo, CAR/CXCR5 T cells accumulated with vRNA^+^ in lymphoid follicular-like structures and were detected in direct contact with vRNA^+^ cells. The CAR/CXCR5 T cells persisted in peripheral blood and secondary lymphoid tissues through the end of the study at 28 DPI. Despite the presence of CAR/CXCR5 T cells, we did not detect reductions of VLs in treated animals. The in situ analysis of HIV vRNA^+^ cells and CAR/CXCR5 T cells in spleen led us to discern a likely mechanism contributing to sustained VLs detected in treated animals; we surmise that HIV infection was spread by HIV-infected CD4 T cells, including a subset of CD4^+^ CAR/CXCR5 T cells, that were present in our product. It is perhaps unsurprising that a subset of our CAR/CXCR5 T cells were susceptible to HIV infection, given that the CAR/CXCR5 T-cell product is composed of both CD4^+^ and CD8^+^ T cells and that CD4 is the primary entry receptor for HIV viral entry. However, in SIV-infected rhesus macaques, we found that only 1 in 689 CAR/CXR5 T cells was SIV vRNA^+^ postinfusion.^10^ It should be noted that while the HIV-specific CD4-MBL-CAR used in this study contains a CD4 domain, this CAR does not act as an entry receptor for HIV, meaning that CAR expression was not responsible for HIV infection of the CAR/CXCR5 T cells.^7^ These findings suggest that our HIV-targeting CAR/CXCR5 T cells might be improved by equipping them with HIV resistance, perhaps by knocking out CCR5.^58–60^^,^^62^^,^^63^ Indeed, in a study by Maldini et al,^29^ it was only after HIV-targeting CAR T cells were made resistant to infection that differences in HIV VLs were detected in treated versus control animals.

In this study, the majority of infused CAR/CXCR5 T cells had a T_EM_ or T_TEM_ phenotype. Ideally, the CAR T cells would have a less differentiated phenotype, as T_EM_ cells are associated with increased exhaustion and reduced clinical responses to CAR T-cell treatment.^61^ Moreover, the CAR/CXCR5 T cells may have been exhausted due to proliferation in response to uncontrolled HIV viremia rather than phenotype alone.^29^^,^^30^ Therefore, the development of CAR/CXCR5 T cells that are less susceptible to exhaustion may improve efficacy. Future studies could explore several means of reducing exhaustion levels. Further engineering could be employed to disrupt PD-1 expression or other inhibitory molecules in CAR T cells.^64^^,^^65^ Cell culture conditions of CAR/CXCR5 T cells could be modified to prevent differentiation and thereby exhaustion.^66^ The co-stimulatory domain of the CAR molecule could also be changed from CD28 to 4-1BB, as CAR T cells with 4-1BB costimulatory domains present a more undifferentiated phenotype and greater in vivo persistence.^9^

Although the CAR/CXCR5 T cells did not suppress HIV VLs in actively infected hDRAGA mice, we did show that ART can suppress VLs in this model. These results further support the use of HIV-infected hDRAGA mice as a model for testing novel therapeutics for HIV. Future studies are warranted where CAR/CXCR5 T-cell therapies are tested following ART suppression of virus to better recapitulate clinical trial conditions.

In conclusion, the current study supports the use of the hDRAGA mouse model for HIV immunotherapeutic studies. Additional studies to improve HIV-specific immunotherapies using this model are warranted, including extending the overall study duration to determine the long-term potential of these therapies in hDRAGA mice. DRAGA mice may also be a valuable platform for studying non-immune-based therapies for HIV as ART was shown to be successful in reducing VLs in these animals. This study also supports further investigation into the potential for DRAGA mice to recapitulate therapeutic trials for other disease states.

## Supplementary Material

vkaf185_Supplementary_Data

## Data Availability

The data underlying this article will be shared on reasonable request to the corresponding author.
