# Supplementary material for: Humanized DRAGA mice are a valuable model to study novel immunotherapies for HIV-1
Source: J Immunol. 2025 Aug 13;214(11):3017–27. doi: 10.1093/jimmun/vkaf185 (PMC12646065; doi:10.1093/jimmun/vkaf185)
Supplement: vkaf185_Supplementary_Data [file vkaf185_supplementary_data.zip › Supplemental Data (1) (1).pdf]

Supplemental data

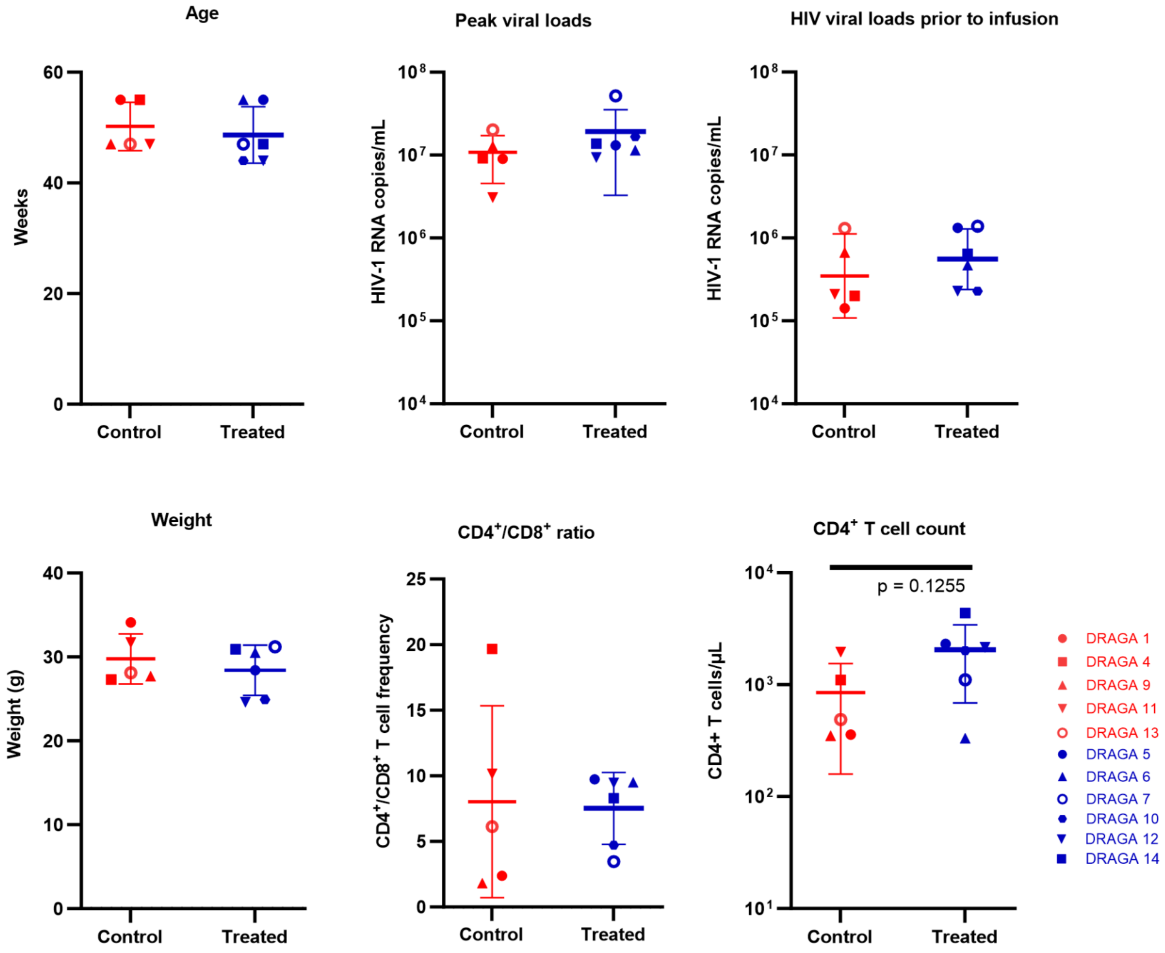

Supplementary Figure 1. Animal allocation considerations before CAR/CXCR5-T cell infusion. Criteria of control and treated animals included age, the peak of plasma HIV RNA viral loads, levels of plasma HIV RNA viral loads before infusion of transduced cell products, weight, human $CD4^+/CD8^+$ T cell ratios, and human $CD4^+$ T cell counts. *P*-values were determined by the two-tailed Mann–Whitney U test. Each square or circle represents an individual mouse. Red = control (untreated) mice; blue = treated mice. The bar and error represent mean ± SD or geometric mean with 95% CI (for $CD4^+$ T cell count).

Supplementary Table I. HIV-1 plasma viral loads in treated and control animals post-cell infusion.

Day(s) post-infusion	Control animals (x 10^5 copies/mL)							Treated animals (x 10^5 copies/mL)							
	**1**	**4**	**9**	**11**	**13**	**Mean**	**SD**	**5**	**6**	**7**	**10**	**12**	**14**	**Mean**	**SD**
0	1.42	2.00	6.66	2.09	13.00	5.03	4.93	13.20	4.70	13.90	2.29	2.29	6.44	7.14	5.21
6	11.30	11.50	21.80	13.10	43.80	20.30	13.80	30.00	17.40	20.10	19.00	12.80	11.50	18.50	6.70
14	13.00	5.42	6.22			8.21	4.16	12.80	7.96	10.40	2.19			8.35	4.55
28	6.16					6.16		33.5	14.7					24.1	13.3
